# A consensus on the diagnosis and treatment of acromegaly complications

**DOI:** 10.1007/s11102-012-0420-x

**Published:** 2012-08-18

**Authors:** S. Melmed, F. F. Casanueva, A. Klibanski, M. D. Bronstein, P. Chanson, S. W. Lamberts, C. J. Strasburger, J. A. H. Wass, A. Giustina

**Affiliations:** 1 Department of Medicine, Cedars-Sinai Medical Center, 8700 Beverly Blvd., Room 2015, Los Angeles, CA 90048 USA; 2 Division of Endocrinology CHUS, Department of Medicine, Santiago de Compostela University, Santiago de Compostela, Spain; 3 Neuroendocrine Unit, Massachusetts General Hospital and Harvard Medical School, Boston, MA USA; 4 Neuroendocrine Unit, Division of Endocrinology and Metabolism, University of Sao Paulo Medical School, Sao Paulo, Brazil; 5 Faculté de Médecine, Université Paris Sud, Orsay, France; 6 AP-HP, Hôpital Bicêtre, Service d’Endocrinologie et des Maladies de la Reproduction, Le Kremlin-Bicêtre, France; 7 Institut National de la Santé et de la Recherche Médicale, U693, Le Kremlin Bicêtre, France; 8 Department of Internal Medicine, Division of Endocrinology, Erasmus Medical Centre, Rotterdam, The Netherlands; 9 Department of Endocrinology, Charite Campus Mitte, Berlin, Germany; 10 Department of Endocrinology, Oxford Centre of Diabetes, Endocrinology & Metabolism, Churchill Hospital, Oxford, UK; 11 Department of Medical and Surgical Sciences, University of Brescia, Brescia, Italy

**Keywords:** Acromegaly, Consensus, Complications, Diagnosis, Treatment

## Abstract

In March 2011, the Acromegaly Consensus Group met to revise and update the guidelines on the diagnosis and treatment of acromegaly complications. The meeting was sponsored by the Pituitary Society and the European Neuroendocrinology Association and included experts skilled in the management of acromegaly. Complications considered included cardiovascular, endocrine and metabolic, sleep apnea, bone diseases, and mortality. Outcomes in selected, related clinical conditions were also considered, and included pregnancy, familial acromegaly and invasive macroadenomas. The need for a new disease staging model was considered, and design of such a tool was proposed.

## Introduction

Since 2000, several consensus documents have been published on various aspects of acromegaly management [1–7]. In 2003 a consensus on the diagnosis and treatment of acromegaly complications was published [3], and in March 2011, the Acromegaly Consensus Group that had produced these documents met to revise and update guidelines on acromegaly complications. The meeting was sponsored by the Pituitary Society and the European Neuroendocrinology Association and included experts skilled in acromegaly management.

Patients with acromegaly have a considerable burden of complications and co-existing illnesses, and factors contributing to increased mortality in acromegaly include higher prevalence of hypertension, hyperglycemia or diabetes, cardiomyopathy and sleep apnea [8]. The diagnosis and management of complications of disease are therefore critical for assuring a favorable long-term outcome for this chronic illness. Current comorbidity issues were discussed by the group and recommendations made for updating the 2003 guidelines.

Recommendations were graded, based on the GRADE system [9, 10], depending on the quality of evidence as very low quality (VLQ; expert opinion with one or a small number of small uncontrolled studies in support), low quality (LQ; large series of small uncontrolled studies), moderate quality (MQ; one or a small number of large uncontrolled studies or meta-analyses), or high quality (HQ; controlled studies or large series of large uncontrolled studies with sufficiently long follow-up). Recommendations were classed as discretionary recommendations (DR) if based on VLQ or LQ evidence, and as strong recommendations (SR) if based on MQ and HQ evidence.

## Cardiovascular complications

Hypertension is highly prevalent, occurring in more than 40 % of patients with acromegaly, and early diagnosis and early aggressive treatment of elevated blood pressure is important irrespective of which acromegaly treatment is employed (HQ) [11–17]. Hypertension in patients with acromegaly is usually mild and readily treated with anti-hypertensive drugs (VLQ) [3]. The choice of treatment for hypertension should be similar to that in non-acromegaly patients (DR). The effect of different medical treatments for acromegaly on hypertension is as yet unclear (LQ) [15, 18–21]. Sleep apnea, which is present in most patients with acromegaly, exacerbates hypertension (LQ) [22].

Cardiomyopathy is present in most patients with acromegaly, and baseline echocardiogram is indicated. Arrhythmia is rarely a significant clinical challenge in acromegaly (MQ) [16, 23]. Treatment of acromegaly improves early and intermediate stage myocardial hypertrophy and cardiac dysfunction (HQ) [24–26]. Improvement depends on age, presence of hypertension and duration of the disease (LQ). Recent data (published after the consensus meeting) show that cabergoline does not aggravate prevalence or incidence of valve regurgitation and remodeling [27], which are increased in acromegaly (MQ) [28, 29]. Furthermore, somatostatin receptor ligands (SRLs) may cause asymptomatic bradycardia (LQ) [30]. Rigorous clinical outcome measures (and not only biochemical goals) should be addressed in research studies.

The following routine baseline assessments are therefore required for patients with acromegaly: electrocardiogram (ECG), echocardiogram, blood pressure measurement, and the Epworth scale or sleep study for sleep apnea (SR) (Table 1). These patients also require assessment of the peripheral arterial system. Particularly in gigantism, there is a need for assessment of vascular disease including peripheral venous disease (DR).

**Table 1:** Assessment of acromegaly complications at diagnosis and during long-term monitoring

Diagnosis	During long-term follow-up
Blood pressure measurement	Every 6 months or when change of treatment (if hypertensive)
Echocardiography	Annually
ECG	Annually
Epworth scale or sleep study	Annually
Echo Doppler of peripheral arterial and venous system	Annually particularly in gigantism
OGTT	Fasting blood glucose every 6 months (particularly in uncontrolled disease and during SRL therapy); HbA_1c_ every 6 months if diabetes present
Total testosterone, SHBG and prolactin (males)	Annually (free testosterone when doubts in interpretation of total testosterone)
LH, FSH, 17β-estradiol and prolactin (females)	Annually (or when pregnancy is desired)
AcroQoL	Annually
DEXA	Every 2 years if patient with osteopenia/osteoporosis
Thoracic and lumbar spine X-ray	Every 2–3 years if osteoporosis risk factors, kyphosis or symptoms
Colonoscopy	Every 10 years (more frequently if IGF-I remains persistently elevated or if abnormal colonoscopy or family history of colonic cancer)
Genetic screening for markers of familial acromegaly (if suspicion)	

*ECG* electrocardiography, *OGTT* oral glucose tolerance test, *SRL* somatostatin receptor ligand, *HbA*_*1c*_ glycated hemoglobin, *SHBG* sex hormone-binding globulin, *LH* luteinizing hormone, *FSH* follicle-stimulating hormone, *AcroQoL* Acromegaly Quality of Life Questionnaire, *DEXA* dual-energy X-ray absorptiometry, *IGF-I* insulin-like growth factor-I

## Endocrine and metabolic complications

### Diabetes

Diabetes occurs more frequently in acromegaly patients than in the general population and is an important predictive factor for increased mortality in patients with acromegaly (HQ) [31, 32]. Treatment of diabetes should be the same in patients with or without acromegaly (DR) [32]. Generally, lowering of growth hormone (GH) levels improves glycemic control and enhances insulin sensitivity (GH is a powerful insulin antagonist), whatever mode of treatment is used (MQ). An exacerbation of carbohydrate intolerance may occur rarely in patients receiving SRLs (MQ) [32–34] (but hyperglycemia occurs more frequently with pasireotide than with other SRLs) [35] and pegvisomant may be considered for treatment of these patients (DR).

### Hypogonadism

Hypogonadism occurs in approximately 50 % of acromegaly patients, but is often reversible unless gonadotroph destruction has occurred (MQ) [36, 37]. Biochemical diagnosis in males may be difficult, due to low sex hormone-binding globulin levels (making interpretation of total testosterone values more challenging) (LQ) [37]. In these circumstances, assessment of clinical symptoms and bioavailable testosterone are important for diagnosis (DR) (Table 1). Concomitant hyperprolactinemia should be considered as a cause of hypogonadism (MQ). Treatment indications for hypogonadism in acromegaly, and the specific therapy used, should be similar to the non-acromegaly population (DR). In women, hypogonadism should be assessed carefully and treated appropriately, especially if fertility is desired (SR) [36].

### Sleep apnea

Sleep apnea is currently under-assessed, and the prevalence is high (up to 70 %) in newly diagnosed patients with acromegaly (MQ) [22, 38, 39]. Therefore, every patient should have a careful symptomatic assessment (e.g., by Epworth score), and if necessary laboratory assessment, for sleep apnea at the time of diagnosis, in collaboration with a respiratory physician (SR).

Despite successful acromegaly treatment, sleep apnea does not consistently resolve (MQ), so post-treatment evaluation is essential (SR) [22, 40–43]. Every effort should be made to improve compliance with prescribed treatments including continuous positive air pressure device and other devices (e.g., with appropriate measures tailored to patients with acromegaly, for example, specialized mouth pieces for use during sleep). Consultation with maxillo-facial surgeons is advised, and if necessary, elective surgery should be undertaken.

## Other comorbidities and mortality

### Quality of life

Quality of life (QoL) is an important outcome measure in acromegaly [44]. Tools for measuring QoL in patients with acromegaly (e.g., Acromegaly Quality of Life Questionnaire [AcroQoL]) are readily available and should be considered in clinical practice (MQ) (Table 1) [44, 45]. QoL is impaired by GH-excess and GH-deficiency, and therefore, when treating patients, it is important to recognize induction of GH-deficiency, particularly in patients who have undergone previous radiation therapy, as this can have further deleterious effects on subsequent QoL and other parameters (DR) [46].

### Bones and joints

Arthropathy affects approximately 75 % of acromegaly patients [47]. Any joint may be affected (large joints, small joints and vertebrae), and range from osteoarthritis to arthralgia to fractures (HQ) [48, 49]. Bony overgrowth and soft-tissue swelling may also lead to nerve entrapment. Such anatomical abnormalities may result in a delay to surgical intervention for acromegaly (LQ). Disability due to acromegaly is probably under-estimated and can affect many aspects of activities of daily living (MQ) [50–52]. Early diagnosis of acromegaly is required to ensure early and aggressive treatment to reduce arthropathy risk, because joint and cartilage changes are irreversible (SR) [53, 54].

Vertebral fractures have been reported to occur in acromegaly patients independently of bone mineral density (BMD) (MQ) [49, 55–57]. Bone size may affect BMD measurement, and the utility of dual-energy X-ray absorptiometry for screening for abnormalities in the absence of size correction is not well documented (LQ). Thoracic and lumbar spine X-ray may be useful for diagnosing spinal deformities, particularly in symptomatic patients (DR) (Table 1). Patients with acromegaly should be evaluated for osteoporosis risk factors including vitamin D deficiency, inadequate calcium intake, serum calcium to assess hyperparathyroidism, glucocorticoid over-replacement and hypogonadism (SR).

### Colon polyps

Acromegaly is associated with increased colon polyps and may be associated with increased risk of colorectal cancer, but not cancer mortality (MQ) [58]. Colon length may increase during acromegaly resulting in increased mucosal folds (dolichocolon) (LQ) [59, 60]. It is recommended that a screening colonoscopy be carried out at diagnosis in adults (SR). If the colonoscopy is negative, then patients should be screened similarly to the general population, especially if insulin-like growth factor-I (IGF-I) levels are normalized (DR) (Table 1) [59]. If IGF-I remains persistently elevated, more frequent screening is recommended (SR). If colonoscopy is abnormal, follow-up and screening should be in accordance with clinical guidelines [59–61].

### Mortality

Uncontrolled acromegaly is associated with increased mortality (HQ) [62, 63]. Co-existing adrenal insufficiency and its therapy may have an impact on mortality (LQ). In comparison with other forms of treatment aimed at controlling GH hypersecretion, conventional radiation therapy is specifically associated with increased overall mortality (MQ) [20, 31, 64]. Radiation therapy increases risk of hypopituitarism that leads to higher mortality; including cerebrovascular disease (MQ) [64]. There is a need for more data on mortality outcomes in patients receiving stereotactic radiation therapy, which will become available as more patients are followed-up in the long term.

Minimal dose of glucocorticoids required for efficacy in hypopituitarism should be used, as over-replacement of glucocorticoids can have a negative impact on mortality (LQ). Attention to the GH status is important when using glucocorticoid replacement therapy because higher replacement doses may be required when GH therapy is given (DR) [64].

Improved medical therapy is recommended to control GH/IGF-I levels and improve mortality (SR) [31, 64, 65]. Gonadal, thyroid and GH deficiencies should also be assessed and properly replaced, when indicated (SR).

## Treatment outcomes in selected clinical conditions

### Pregnancy

In women with acromegaly who wish to become pregnant, pituitary function should be assessed and surgical or medical treatment initiated as indicated (SR). There are few data (animal or human) on the safety of pegvisomant in pregnancy (VLQ) [66, 67].

Unless clinically indicated, pharmacological agents should be discontinued on confirmation of pregnancy (SR). In some pregnant women, IGF-I levels remain within the normal range (for non-pregnant women), even in uncontrolled patients, and then increase after delivery. Biochemical assessment of disease activity is of limited value because of placental production of GH and IGF-I (LQ) [68, 69]. There is a need to encourage reporting of outcomes in medically treated pregnant women with acromegaly.

### Familial acromegaly

Familial acromegaly is generally diagnosed at an earlier age than non-familial acromegaly [70], and young patients (<30 years old) with aggressive acromegaly, or individuals with a family member who has a pituitary tumor, should be considered for genetic screening for markers of familial acromegaly (DR). Carney complex, familial isolated pituitary adenoma (FIPA) and multiple endocrine neoplasia type 1 should be considered (DR) [71]. An increased awareness that FIPA is associated with mutations in the aryl hydrocarbon receptor interacting protein (AIP) gene is required [70, 72–74].

### Invasive macroadenomas

Access to multimodal treatment and involvement of a multi-disciplinary team are needed in patients with invasive macroadenomas (HQ). If visual fields are impaired, immediate surgery is typically the first line therapy (HQ). Pre-treatment with SRL may improve subsequent surgical outcomes (MQ) [75–77]. Surgical debulking may improve subsequent medical treatment outcomes (LQ) [78–80]. Radiation therapy may continue to have a place in the acromegaly treatment algorithm (MQ) [81–84]. The choice of treatment strategy should include consideration of comorbidities, the projected length of time for hormone normalization, therapy side-effects, pituitary function and cost-benefit of the indicated strategy (DR).

## Disease staging

### Biochemical control

Over the past decade, biochemical criteria defining ‘control’ of disease activity have become increasingly rigorous [4, 85]. The most recent definitions of active disease (random GH ≥1 ng/ml; nadir GH after oral glucose tolerance test (OGTT) ≥0.4 ng/ml; and elevated IGF-I for age) and optimal disease control (random GH <1 ng/ml when treated with SRLs; nadir GH after OGTT <0.4 ng/ml after surgery; and age-normalized IGF-I levels for all treatments and as a unique parameter with pegvisomant usage) remain valid (SR) [4, 86]. However, the definition for optimal disease control comes with the caveat that assay-specific normative data for post-OGTT nadir are required (DR).

### Tumor control

The role of tumor characteristics and of optimal tumor growth control has not been fully agreed by consensus. Factors proposed to be associated with disease persistence after surgery and/or a sub-optimal response to medical therapy include:

- Younger age at diagnosis (LQ) [87–89].
- High expression of tumor aggression markers like Ki67, p53 and pituitary tumor transforming gene (PTTG) (LQ) [90–93].
- Sparsely granulated adenomas [94] and hyper-intense imaging on T2 weighted magnetic resonance imaging (MQ) [94–98].
- Very large adenomas and actively growing tumors (MQ) [99–103].
- No previous radiation therapy, especially during pegvisomant therapy (LQ) [6, 104–106].
- Previous, sub-optimal response to SRL therapy (LQ) [107–112].
- High GH and IGF-I levels during long-term follow-up (HQ) [102, 103, 107, 108, 110, 113–115].
- Larger tumor remnants after surgery (MQ) [114, 116].

### The need for a staging tool?

A new model for describing overall patient assessment before and during treatment would be valuable. The rationale supporting the need for such a model is the requirement to integrate a number of clinical metrics as well as information on pituitary tumor characteristics with the currently used biochemical markers. Ideally, such a model should include data on:

- Clinical symptoms.
- Associated clinical signs and comorbidities.
- GH level.
- IGF-I level.
- Tumor size.

## Conclusions

Effective management of acromegaly complications will lead to decreased morbidity and mortality, and improved QoL. Comprehensive multimodal acromegaly management should integrate biochemical monitoring, careful assessment of tumor behavior and clinical features, and comorbidities.
